# Pulled in or pushed out? Understanding the complexities of motivation for alternative therapies use in Ghana

**DOI:** 10.3402/qhw.v11.29667

**Published:** 2016-03-24

**Authors:** Razak Mohammed Gyasi, Felix Asante, Joseph Yaw Yeboah, Kabila Abass, Charlotte Monica Mensah, Lawrencia Pokuah Siaw

**Affiliations:** 1 Department of Sociology and Social Policy, Lingnan University, Tuen Mun, New Territories, Hong Kong SAR; 2 Department of Geography and Rural Development, Kwame Nkrumah University of Science and Technology, Kumasi, Ghana

**Keywords:** a posteriori, Ashanti Region, cultural belief, health-seeking behaviour, pull factors, traditional medicine

## Abstract

The impact of strong cultural beliefs on specific reasons for traditional medicine (TRM) use among individuals and populations has long been advanced in health care and spatio-medical literature. Yet, little has been done in Ghana and the Ashanti Region in particular to bring out the precise “pull” and “push” relative influences on TRM utilization. With a qualitative research approach involving rural and urban character, the study explored health beliefs and motivations for TRM use in Kumasi Metropolis and Sekyere South District, Ghana. The study draws on data from 36 in-depth interviews with adults, selected through theoretical sampling. We used the *a posteriori* inductive reduction model to derive broad themes and subthemes. The “pull factors”—perceived benefits in TRM use *vis-à-vis* the “push factors”—perceived poor services of the biomedical treatments contributed to the growing trends in TRM use. The result however indicates that the “pull factors,” *viz*.—personal health beliefs, desire to take control of one's health, perceived efficacy, and safety of various modalities of TRM—were stronger in shaping TRM use. Poor access to conventional medicine accounted for the differences in TRM use between rural and urban areas. Understanding the treatment and health-seeking behaviour of a cultural-related group is critical for developing and sustaining traditional therapy in Ghana.

This paper attempts to advance our understanding about the convolutions of personal health beliefs and related motivations for the use of traditional health care services in a Ghanaian rural and urban context. The impact of belief systems and specific reasons for traditional medicine (TRM) use among populations has long been advanced in copious health care and spatio-medical literature. Yet, not much has been done generally in Ghana and the Ashanti Region in particular to bring out the precise “push” and “pull” relative influences in TRM utilization. Indeed, no current uniform and directional analysis of TRM use has been documented. Besides, there is an overt knowledge gap on the in-depth, qualitative discussion about beliefs and the associated motivations for TRM use, given the complex normative and perceived nature of the subject. Most studies remain parsimonious and have focused severally on purely quantitative and much rigorous statistical analysis in trying to explain belief systems and their connection to TRM use (Chao, Wade, Kronenberg, Kalmuss, & Cushman, 2006; Lövgren, Wilde-Larsson, Hök, Leveälahti, & Tishelman, 2011; Sirois, 2008a). The findings of these studies cannot accurately reflect the reality and therefore remain confounding. It is in an attempt to bridge this research lacuna that this study emerges, using data from TRM up-takers in the Ashanti Region of Ghana.

Acute and chronic anguish associated with ill health have a strong effect on health care and health-seeking behaviour. In the management of disease afflictions, individuals and communities may resort to a wide range of medical practitioners and medication modalities, often combining different TRM and biomedical treatments (Arcury et al., 2006; Bell et al., 2006; Egede, Ye, Zheng, & Silverstein, 2002; Garrow & Egede, 2006; Gyasi, Asante, et al., 2015; Gyasi, Mensah, & Siaw, 2015; Gyasi, Siaw, & Mensah, 2015; Spinks, Johnston, & Hollingsworth, 2014). TRM, involving a diversity of approaches, systems, practices, and theories of medicine that fall outside the realm of conventional modern medicine, has carved an enviable niche for itself in the field of health promotion, disease management, and prevention the world over. In most parts of sub-Saharan Africa, TRM remains the primary care medium for the majority, assuming the role of complementary and alternative or substitute to the conventional scientific medicine. In Ghana, traditional systems of medicine are recognized as an integral part of the cultural and a traditional system of the people (Gyasi, Mensah, Adjei, & Agyemang, 2011).

Studies in developed and developing countries have reported variations in the prevalence of TRM acceptance and up-take, ranging from 16 to 90% with an increasing trend (Awad & Al-Shaye, 2014; Demirci & Altunay, 2014; Faith, Thorburn, & Tippens, 2013; Hwang, Han, Yoo, & Kim, 2014; Kretchy, Owusu-Daaku, & Danquah, 2014; Gyasi, Siaw, et al., 2015; Gyasi, Tagoe-Darko, & Mensah, 2013). Despite the current dispensation, influx, and remarkable advances in the classic orthodox medical system, TRM continues to play a momentous part in the health care system of nearly every nation (Eisenberg et al., 1998; Schimpff, 1997; Yeo et al., 2005). Typically, more people turn to the complementary medicine on a daily bases for their medical and spiritual problems (Adib-Hajbagherya & Hoseinian, 2014; Gyasi, Siaw, et al., 2015).

The belief paradigm that surrounds motivation for the choice and use of TRM has been elucidated amply in various studies. One school of thought advocates for the “pull” subtleties which apparently constitute the perceived benefits that induce individuals to consume TRM. A desire to take an active role in one's own health and holistic health beliefs are among the more often cited “pull” variables (Sirois, 2008b; Sirois & Gick, 2002; Verhoef, Balneaves, Boon, & Vroegindewey, 2005). Others find TRM attractive because it is in agreement with their personal values, religious backgrounds, and health philosophies (Furnham & Forey, 1994; Osamor & Owumi, 2010). The safety and efficacy of TRM in dealing with spiritual and psychological conditions is also reckoned. Conversely, negative aspects of conventional treatments that potentially repel patients make up the “push” dynamics of TRM use. Some patients use TRM because they are dissatisfied and feel uncomfortable with modern medicines that are perceived to be ineffective for “tropical” and neglected diseases, expensive, or have unpleasant side effects (Boon, Brown, Gavin, & Westlake, 2003; Shmueli & Shuval, 2006; Sirois & Purc-Stephenson, 2008) partly due to chemical infestation.

Various studies have debated about the “push” away from conventional treatments and “pull” into TRM factors. However, there is a dearth of qualitative research articulating the strength of influence amassed from each set of factors in the Ghanaian landscape. The purpose of this study is to explore the perspectives of the adult Ghanaian population regarding the motivations for TRM utilization.

## Methods

### Study design

This study investigated the belief systems and the associated reasons or motivations for TRM use among the adult population in the Kumasi Metropolis and Sekyere South District of Ashanti Region, Ghana. It is crucial to gain an in-depth understanding of why most people, both rural and urban dwellers, continue to utilize TRM in the context of modern day advancements in medical technology. Data for this study were gathered as part of a larger research of the analysis of TRM utilization in Ghana. Involving TRM users from diverse socio-economic and cultural landscapes, the study espoused a qualitative research approach (Creswell & Design, 2008). The study relied on an interpretivist paradigm, relativist ontology and subjectivist epistemology (Angen, 2000; Berger & Luckmann, 1967) with the understanding that these approaches to research proffer a better way of tapping into natural real-life experiences of TRM users with respect to the belief milieus and motivations for TRM consumption. These methods ensure an adequate discourse between the researchers and those with whom they interact in order to construct a collaborative meaningful reality (Guba & Lincoln, 1994; Morgan, 2007).

### Sample selection and data collection procedure

The study draws on data from 36 community members who had used any form of TRM and/or had accessed the services of traditional medical practitioners (TMPs) in the last 12 months prior to the time of the interview. To bring the real situation into being regarding TRM use and to curb incidence of research bias, rural-urban character, *vis-à-vis* sex dimensions were critically taken into consideration during the sampling processes. Altogether, 19 respondents were taken from the rural Sekyere South District and 17 were selected from the Kumasi Metropolis, an urban community. This selection approach was adopted arbitrarily regardless of any parameters such as the size of the target population and/or the accessible population of the study following Barbour (2001). However, a greater share was given to the rural setting because most of the rural residents are more likely to use traditional medical services than their urban counterparts (Adams, Sibbritt, & Lui, 2011). Also, half of the total sample size constituted females. This was done to play away the possible incidence of sex differences in the TRM consumption. This sample size, selected through a non-probability theoretical and purposive sampling technique, was deemed adequate for qualitative, descriptive, and model building purposes. Community members were asked to indicate their TRM use status regarding the definition for TRM use. Individuals in the study communities who met the inclusion criteria were recruited to partake in the study.

The field work was conducted between March and June 2013. A face-to-face in-depth interview guide was used as the data collection instrument to obtain pertinent data from the respondents. Those who were willing to participate but could not take time out immediately were asked to provide their own sober moments for the conduct of the interview. Interviews were conducted in the homes of the respondents after they had scheduled a convenient day and time for it. On the average, each interview lasted approximately 35 min. With permission from the interviewees, the interviews were recorded digitally for word-perfect transcription into word files and analysis. In accordance with ethical requirements, all participants were offered the option of reviewing and possibly amending aspects of the transcript to reflect the original information they provided. A range of techniques was used to check the consistency of information obtained and also provide detailed understanding of the health-seeking behaviour pattern of the respondents. As a result, individuals below the statutory age of 18 years were excluded from the survey (Republican Constitution of Ghana, 1992).

### Ethical issues and study protocol

The study was approved by the Committee on Human Research Publication and Ethics (CHRPE), School of Medical Sciences at Kwame Nkrumah University of Science and Technology (KNUST) and Komfo Anokye Teaching Hospital, Kumasi (CHRPE/AP/260/14). All respondents earmarked for the survey were approached and systematically briefed about the purposes of the study. Their personal consent was sought as detailed by the principles of the Declaration of Helsinki (World Medical Association, 1978). Participation was voluntary and the participants reserved the right to withdraw from the interview at any time or decline providing responses to certain questions as and when they deemed fit without any query whatsoever.

### Data analysis

The qualitative data obtained from the perspectives of the various categories of respondents in relation to personal health beliefs and perceptions regarding motivation for TRM use were analysed thematically by comparing the responses in order to identify common trends, similarities, and contrasts through the application of Grounded Theorizing Approach (Guba & Lincoln, 1994). We applied the *a posteriori* inductive reduction methodology to develop broad and consistent themes (Barbour, 2001; Bryman & Burgess, 1994; Glaser & Strauss, 1967). Any explanations or theories that emerged were derived from the dataset itself rather than from the researchers’ prior theoretical perspective. The bone of contention was the specific motivation for TRM use which was then categorized under “pull factors” and “push factors.” Another subject that emerged was the challenges respondents face in their bid to utilize TRM. Specific normative and subjective views from the perspectives of study participants have been presented through direct quotations and the emerging conceptual categories merged to form the larger thematic constructs of our schematic model.

## Findings

Findings of the study were deduced from the responses of the 36 adults (recruited for the survey) from different geographical backgrounds, rural (*n*=19; 53%) and urban (*n*=17; 47%), with an evenly split sex differentials (*n*=18; 50% females). Age of respondents ranged from 25 to 74 years. Respondents were affiliated to a wide range of religious denominations with the majority being Christians (*n*=21; 58%). Others professed to Islamic (*n*=11; 31%) and African traditional (*n*=4; 11%) religious beliefs. A few were graduates from tertiary institutions (*n*=6; 17%) and were employed in the public service sector. Almost half (*n*=16; 44%) of the respondents were widows, widowers, separated, or divorced and had retired from active economic ventures. The study revealed distinguished differences in the perceptions and views of respondents regarding reasons for TRM use, particularly between the rural and urban settings. However, three major themes emerged from the analytical output, *viz*., the benefits one derives from choosing and using TRM (“pull factors”); the perceived bottlenecks, disadvantages, and dissatisfaction with conventional health care system and practices (“push factors”); and challenges besetting development and use of TRM. The reasons to use TRM fell into one of the two aforesaid broad subcategories or themes.

### “Pull in” subtleties

A number of reasons explicate patients’ choice and use of TRM and indigenous therapies. Through in-depth interviews, respondents indicated a range of perceptions and beliefs about TRM which in one way or another, influenced prevalence and pattern of TRM use. TRM was accessed for all areas of health care—preventive, curative, and rehabilitative—by the respondents. The study participants described various “pull factors” which to a greater extent influenced their use of TRM. These constituted a set of positive mechanisms that were derived from consuming various aspects of TRM and therefore exerted a pulling effect towards TRM use. Generally, these were engulfed by the cultural belief systems of respondents and their perceptions about TRM. The majority of them expressed that part of their motivation for using TRM was due to it being more natural, involving no or little chemicals, and therefore, having minimal side effects. Other respondents also poised that natural things are generally without chemicals and therefore are safe to use. Emphasis was placed on the importance of consuming leafy vegetables not only for their dietary or nutritional value, but also their medicinal effects as opposed to processed and chemicalized medicines. These standpoints were notable among both rural and urban participants and across both sexes as the following quotes depict:

Yeah … it's true. Medicinal plants or herbs are natural and pure. Natural plants are free from chemicals unlike the white man's drugs. Chemicals they put in medicines are dangerous; they could have long-term devastating and degenerative effects on our body system. I don't like those drugs because I don't want to put chemicals into my body. That's why I always go for herbs anytime I'm ill. They (herbal medicines) are safe. [A 43-year-old woman, Urban]

I think herbal preparations are gentle and less powerful on our bodies. It's flexible too that's why we're free to take any dose without harm. I hope you know that vegetables and all leaves are herbal medicines. Then tell me why you can take any quantity of them at any time but will cause no problem for you? Look at “kontomire” (leaves of the cocoyam or taro plant), “kwawunsusaa” (solanum tovum), onions, cabbage, carrots, lettuce, pawpaw leaves … they're all herbal medicines for certain diseases but harmless. They even provide enough blood for the body and help in digestion. Isn't it true? I can't say they [herbal medicines] are all good for our health. They're better than prescribed drugs from hospitals and pharmacy shops in terms of safety. [A 68-year-old man, Rural]

Issues related to efficacy were highlighted several times (32 of 36 interviewees). The respondents felt that TRM treatments and therapies were effective, particularly in handling the perils and the burden of “tropical” and neglected diseases such as malaria, typhoid fever, jaundice, sexually transmitted infections (including HIV/AIDS), infertility, menstrual problems, sexual weakness, piles, cold, influenza, cough, hernia, headache/migraine, stomach/intestinal problems, chronic skin diseases, bone fracture, and arthritis as well as chronic non-communicable diseases including hypertension, cancers, epilepsy, spiritual health, and illness of psychic origin. Respondents endorsed TRM as efficacious and potent as the following excerpts were generated to confirm this assertion:

… I never trusted the healing power of traditional medicines until I was taken to a mental healing centre called Holy Cherubim and Seraphim Healing Church of Ghana at Tepa. I had a mental illness that took me and my family to almost every psychiatric hospital across the country for over eight years. I was made to take all sorts of drugs that made me feel weak and disorganised all the time. The Faith Healer intervened and it took him less than three months to set me free. As you see, I feel good … no more stigma, no more pain, and people respect me now …. [A 32-year-old man, Urban]

That medicine [TRM] is good. My own son fell and broke his leg at school. I took him immediately to a hospital here [I don't want to mention it] for several occasions, the Plaster of Paris was on but the leg kept on swelling and the child was suffering. A colleague teacher recommended someone, a bone setter at Alabaa in Kumasi here. We went together and the child was able to walk after one week. Most aspects of TRM are very potent and effective. My brother, I don't joke with TRM at all. [A 44-year-old teacher, Urban]

Respondents turned to the use of TRM for reasons of easy access and cost-effectiveness. An overwhelming number of study participants (29 of 36 interviews) said they were motivated to rely on TRM because it was readily available and easily accessible; that most aspects of TRM, especially biologically-based modalities could be found at any time without much stress. Examples were given where TRM could be obtained in the backyard or could be purchased at a lower cost. This was a common phenomenon and repeatedly quoted by most of the interviewees. However, unlike the rural participants, the respondents from the urban environment expressed little support to this argument. The following excerpts give further elucidation to this assertion.

Yes, it is not expensive [as compared with hospital]; sometimes you're required to pay only few pesewas to the healer for treatment. Here too you don't also travel far for high lorry fares. They [traditional medical practitioners] are here with us. So, to me, herbal medicines are cool …. [A 33-year-old university student, Urban]

Hahahaha [giggling] … Hmmm … we call it poor man's medicine … “agyenkwa” [literary meaning, saviour]. I think that most people use more of traditional medicines because they're poor people. You see, poor people in the rural communities here cannot afford hospital fees so they go to the herbalists for help. Hospital drugs are always very expensive and they [hospitals] are far away too. Even the health insurance that President Kufour brought does not work to perfection now. Drugs are prescribed for you to buy … it's very expensive to go to hospital. If it's not an emergency, then people will continue to rely on herbal or traditional medicines for all kinds of diseases. I suppose …. [A 62-year-old farmer, Rural]

A major factor that contributed to preference for, and interest in TRM, was the holistic approach and excellent affective behaviour of TMPs. This was a common assertion in the interviews. The argument was wholly based on the belief that the indigenous practitioners were experienced, knew their clientele, and provided technical client-centred treatment. Most participants described the good attitude and excellent human relations of the TMPs towards their clients in the illness episode. From the interviews, two study participants had these to say:

I don't know but I think it's the behaviour and the attitude of the traditional healers that seem to attract most people to go to them for medical and spiritual help. You see, they [traditional healers] have time for every sick person. Some can even let you forget about your sickness or pain and give you full assurance of good treatment before they even apply medicine on you. [A 31-year-old man, Rural]

They [traditional birth attendants] have patience. If you're not able to follow accurately the strict instructions they give you, they don't insult you … embarrass you as some nurses do to us …. [A 26-year-old expectant mother, Urban]

The use of TRM in the restoration of health and well-being was a hallmark of the individual respondents. These findings were validated by the urban respondents interviewed. A university student maintained that using herbal medicine and other aspects of TRM enabled him take a more active part in maintaining his own health. He did not really appreciate why accessing medical treatment and use of his medications should be a rigid process to go through. He held the belief that it is good, easy, and refreshing to be able to sort things out for himself in terms of treatment-seeking routine. He passionately iterated that:

How can you be cured with a treatment regimen you don't even understand? Someone sits somewhere and controls your own health, tells you what you should do … as if he owns your life …. [A 25-year-old man, Urban]

The cultural values and traditions of people determine their psyche which in turn influences their health-seeking behaviour. Some of the study participants maintained that TRM concurs with their religious, cultural, and spiritual beliefs. An old male respondent explained how traditional beliefs have influenced him to use TRM and to access the services of TMPs. The excerpts below confirm this assertion

I earnestly believe in TRM. I know it to be part of my culture and total upbringing. It always yields quite satisfactory results when I use it to treat my illness. It might be psychological, yea …. [A 74-year-old man, Rural]

Some respondents perceived spiritual illness as a reason to seek traditional medical care. There is the belief that certain diseases are caused by spirits. Respondents explained that diseases that are spiritually oriented can only be treated and reversed through spiritual means. To them, Newton's second law of motion—action and reaction are equal and opposite—can conveniently be applied to some spiritual health problems and their solution thereof. This quotation by a middle-aged woman explains further, that:

I wouldn't waste my time at a hospital for doctors to apply their “trial and error” tactics to my spiritual problems. They could only try to put a round peg in a square hole; which cannot fit. They only treat illnesses from the physical point of view. You know, herbalists, spiritualists, and diviners are always the right people for such spiritual problems. They can read, see, and reveal things that are hidden. [A 46-year-old woman, Rural]

Another respondent in the rural setting had this to say:

For sure, the traditional healers treat patients not merely by experience but also by a unique theoretical system which cannot be explained by modern sciences. They can tell the future from today. This is why they're able to cure certain diseases such as epilepsy and mental disorder which are intractable to prescription drugs. [A 72-year-old woman, Urban]

### “Push out” factors

In converse, most respondents attributed increased use of TRM to several problems they have with conventional health care practitioners or aspects of the modern health care system. Some respondents passionately expressed concerns about dissatisfaction with conventional medicine in terms of effectiveness for most of their medical problems such as malaria, excruciating boils, broken bones, mental disorders, and other psychological or spiritual problems, and hence having alternative treatment was prioritized. The following excerpts confirm this claim:

As for me I only go to the hospital for specific medical problems … like check-ups or when I need X-rays or … yeah. Their medicine [prescribed drug] is not good for many of my problems. If you get diseases like piles, boils, fevers and others, hospital cannot help you at all. You'll take all the drugs on this earth but the problem will still be haunting you. It's better to get herbs. [A 22-year-old lady, Urban]

But … doctors do not have the eye to see any “sunsun mu yadee” [spiritual problems]. They'll just be doing trial and error and before you realise your casket is close to you. Isn't it better to rather see a medicine man? [A 56-year-old woman, Rural]

Safety of conventional therapies was a repetitive subject and a major concern to the study sample. Most respondents mentioned that prescribed drugs contain chemicals that may have both momentary and long-term side effects. One respondent noted that:

Many “white man's drugs” have side effects … when you take more of them. I know they add more chemicals to it …. I don't know why but I won't take more to have any problem. I'm always afraid of it … not comfortable taking them because they are not safe For example I daze always when I take Artesunate Amodiaquine for malaria, but Time Herbal Mixture is good for me without any side effects. [A 26-year-old man, Urban]

One area that merited a “push” away from conventional health services was the difficulties associated with accessibility and its concomitant inequities. The study participants juxtaposed the inconvenience of getting appointments with a physician with instant access to TMPs and their services. In an interview, an old lady explained that patients in most cases spend several hours at the health facility to see a physician after paying so much for long distance travel. She went on to lament on the inadequacy of health facilities at her disposal.

Hmmm, my son, it's not because the prescribed drugs will not work for me but … see, you sometimes have to spend the whole day just to see a doctor. When I took my granddaughter to the hospital, we had to wait for so many hours from the morning to late afternoon before we could see the general practitioner. If I go to a herbalist, I can go back in the next one hour time. But it's not their fault too. We have few hospitals in this area and many people go to them. [A 67-year-old woman, Urban]

An area of importance for most people was the attitude of some general practitioners (GP) towards their patients, principally as regards emotional issues. The doctor-patient or nurse-patient relationship is an important one as the doctor and/or nurse interacts with the patient. Past negative experiences with health care professionals—doctors, nurses, midwives—had led to the reluctance to consult GPs and therefore heavy reliance on TRM/TMP. Again, biomedicine is sometimes viewed as distinctly foreign. People consider biomedical practice as a “distant health care system” which does not belong to the local people.

As you see me, I don't want embarrassment but that's the food of most hospital workers, especially the female nurses. Some don't even see you as a fellow human being when you go to them for treatment … they talk to you as if you're a child. For me, orthodox medical system is alien and I'm not comfortable with it. [A 42-year-old man, Rural]

### Challenges of accessing TRM

A number of challenges pertaining to access and use of various forms of TRM, particularly, the herbal-based therapies were observed. One major concern participants shared was linked to the quality control and regulatory mechanisms for herbal medicines. The participants explained that the efficacy and safety of some aspects of TRM cannot be guaranteed because they lack the necessary clinical tests and checks that are requisite to confirm the effectiveness as well as safety.

My brother, these drugs [herbal medicines] can cause us permanent injury and even death. That's my problem. Most of them are not tested and tried. We buy them from peddlers mostly in the open markets and at bus terminals with no idea of how safe they are and the extent to which they can work. We just hope that our problems will be solved but it's only God that cares for us.

Poor packaging and labelling of the herbal medicines were noted as serious bottlenecks. The participants spoke of the herbal preparations having no expiration and dosage indications. This, they concluded could torment the quality of life of individuals and exacerbate the health problems of people. This was mostly asserted by the urban participants who mostly purchase herbal drugs and products from pharmacy shops and street vendors. A respondent lamented that although aspects of TRM are good, if proper quality control measures are not enforced to monitor strictly the preparation and distribution through to the use of herbal medicines; safety of the unsuspecting public is likely to be compromised. The clients therefore cannot derive the best of healing from it.

You see, most of the medicines are not well presented to appeal to us. The drugs might be okay for your problem but you can't be comfortable when taking them. I think the authority in charge should do well to see to improve on the standards and quality of the medicine so that we can be safe

My only problem here is that most of the herbal products do not have expiry dates on them. In this case we can't tell whether or not the drugs are good to use ….

Other respondents emphasized that the low levels of education and training of traditional healers could limit their professionalism and efficiency. One participant mentioned education as an important tool in medicine preparation and administration. He concluded that lack of education may result in contamination of medicines during preparation. It is also not possible to be able to determine, accurately, the chemical and active components of the various plant parts that go into the medicine. Another respondent noted that hygienic conditions and proper environmental upkeep are key in the preparation of medicines. However, most traditional healers practice with less or no education. This raises eyebrows when taking herbal medicines from such healers.

Sometimes people get problems when they take some herbal medicines not just because it's not good but lack of education can even contaminate the medicine in the preparation process

One challenge respondents spoke about was related to the financing of traditional health care. This was directly related to the introduction and implementation of the National Health Insurance Scheme (NHIS) nationwide but excluding the TMPs and their practices. Respondents found it difficult to understand why herbal medicines administered and dispensed at hospitals particularly in the Kumasi Metropolis were not covered under the NHIS and one respondent at Atonsu lamented as follows:

When I went to the Agogo Hospital, the doctor prescribed that I should go to the Herbal Unit for herbal medicine. The man there told me to pay for them before I could take the medicine whilst my NHIS card was active. This is very sad. Discrimination here, discrimination there, discrimination everywhere!!

The concern of “communal healing” or “cure all concepts” was raised as being peculiar to herbal products and remained a challenge in TRM use. Two different perspectives were, however, shared with regard to the use of one medicine for curing multiple ailments. A middle-aged man in the urban community described this as a fallacy and therefore a major problem he had with TRM. “It's difficult for me to understand how one medicine could claim to cure almost all the diseases under this sun.” Another participant noted this was not a problem and tried to explain the phenomenon that a single herbal drug could be prepared from a variety of plants with different plant parts and other natural substances. Each of them may be potent in dealing with a certain disease. He again stated that one disease may show up a number of symptoms. Once the medicine cures the disease, all other symptoms are automatically dealt with.

People will not understand it but I think that's not a problem at all. Once many plants and other things are all put together to make one medicine I will not doubt the medicine being able to cure many diseases. Also, for example, if my constipation causes me to have headache and I found a medicine that cures constipation, the headache will also be cured. Don't you see that? This will appear that the medicine treats both diseases. This's very simple.

## Discussion

The study provides a detailed analysis of the specific factors associated with the motivations to access and use various modalities of TRM among the adult population. Congruent with the findings of previous studies in both economically developed (Lövgren et al., 2011; Sirois & Gick, 2002; Williams, Kitchen, & Eby, 2011) and less developed countries (Gyasi et al., 2011; McLaughlin, Lui, & Adams, 2012; Tabi, Powell, & Hodnicki, 2006; Vickers, Jolly, & Greenfield, 2006), the current study found empirical evidence to suggest that cultural attitudes and personal beliefs and traditions of the people have a strong and complex relationship with motivations for TRM use and the practices of TMPs which goes beyond sociodemographic determinants such as educational levels, professional occupations, and across all age categories in both urban and rural settings in Ghana.

In this perspective, the study reveals that the people are likely to be influenced and motivated by perceived benefits that traditional healers and their medical practices offer clients who present a wide range of medical and spiritual and or psychological problems for assistance. These constitute the “pull variables” for TRM consumption and also validate the study proposition. In consonance with the observation of Sato and Costa-i-Font (2012), the findings present utilization of TRM to reflect the traditional connotations of what actually constitute health and ill health. The holistic nature of TRM is still an important route for its use. The ability of TRM to treat not just an aspect of the being and or a defined disease but a whole being is critical. Unlike the scientific medicine, TRM conveniently deals with both physical and spiritual/psychological/emotional problems towards a “whole health” restoration. The cultural milieu of the local people subsumes TRM use. Good affective behaviour of TMPs and their cordial relationships with their clients pull many people, resulting in a wholesale consumption of TRM. This finding is akin to the observation of other studies which report that patients continue to access TRM partly because healers are socially closer to their clients, understand their language, and also have a good relationship with them (Holst, Wright, Haavik, & Nordeng, 2009; Peltzer, Natalie, Ramlagan, & Fomundam, 2008; Vickers et al., 2006).

The findings suggest that various aspects of TRM are safe. An average individual believes that TRM is safe to use relative to the conventional therapies chiefly because TRM is mostly “natural” with less side effects. Whilst this finding is congruent to some previous studies (Gyasi, Mensah, Yeboah, & Siaw, 2015; Hollyer, Boon, Georgousis, Smith, & Einarson, 2002; Holst et al., 2009), it is inconsistent with other research outputs which reported that TRM is less safe than modern medicines (Addo, 2007; Ernst, 2002; Sato & Costa-i-Font, 2012). This disparity arguably roots from the diversity of individual perceptions and the variety of medications people access in dealing with specifics and/or differences in ill health. People associate the educational standards of TMPs and the general processes of medicine preparation to the safety of use of TRM. As variously cited in both developing and developed countries (see Dog, 2009; Gyasi et al., 2011; Hwang et al., 2014; Mensah & Gyasi, 2012; Sen, Chakraborty, & De, 2011), this study buttresses the effectiveness of TRM particularly in dealing with tropical and neglected health problems. Diseases must be treated through the exact ways by which they emerge. This principle is embedded in TRM practices. Most orthodox medical therapies are not evidence-based; they can only work for either a proportion of patients or an aspect of the body (Chao et al., 2006; Gyasi, Asante, et al., 2015). This potentially repels many people into TRM use.

In the evaluation of health behaviour, values and social factors—spirituality, customs, religious and personal beliefs and philosophies—are critical agents that pull people into TRM utilization. Spirituality and religion have been introduced into the medical realm implying a growing interest in the possible perceived health benefits connected with having a spiritual belief and/or following a religious belief (Kretchy, Owusu-Daaku, & Danquah, 2013; Penman, Oliver, & Harrington, 2009). People might be attracted to TRM use because they hold beliefs that are congruent with TRM practice.

Interestingly, some participants aligned their preference for herbal medicines and other modalities of TRM to personal control. Indeed, the desire to take responsibility for one's own health and well-being and to make their own health care choices is paramount. TRM use grants individuals and families the opportunity and the freedom to choose to shape up one's health. The study found that most respondents from rural areas noted that TRM better serves them in terms of flexibility of access and use as reported by previous studies (Chang, Wallis, Tiralongo, & Wang, 2012; Chao et al., 2006).

The study found that economic milieu which directly or indirectly relates to cost-effectiveness and the ready availability of aspects of TRM in one way or the other influence the rate of TRM up-take. This is consistent with the corpus of previous research findings (Gyasi et al., 2011; Sato & Costa-i-Font, 2012). However, weak influences were generally proffered by the negative and unpleasant aspects of orthodox health services regarding TRM utilization by the adult population, and therefore tend to be supportive in outlook. This was consistent with other studies elsewhere which have reported independently that aspects of modern medicine that push people into TRM use are only an ancillary to the various pull mechanisms (Astin, 1998; McLaughlin et al., 2012; Siahpush, 1999; Sirois & Gick, 2002).

**Figure 1:**
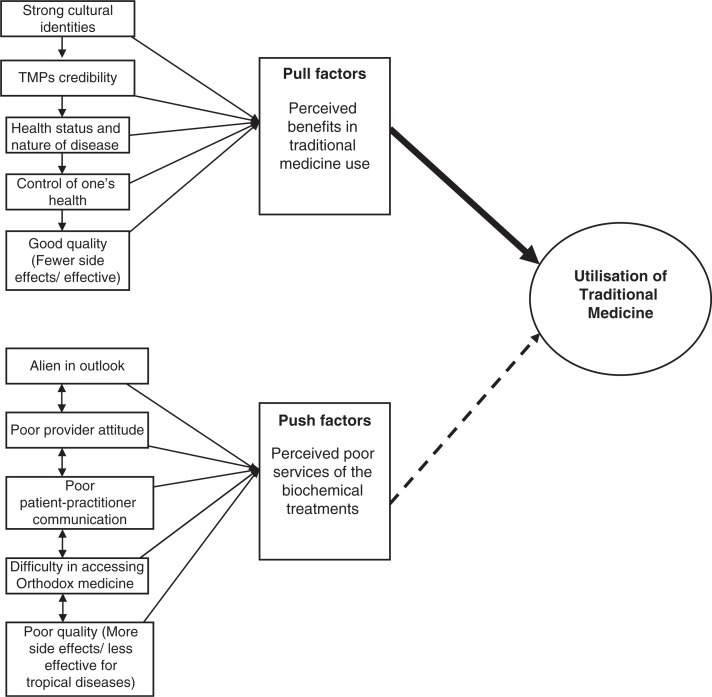
Conceptual model depicting motivation for traditional medicine utilization in Ashanti Region, Ghana. Source: Authors’ construct based on field survey results, 2013.

The study draws attention to a myriad of challenges pertaining to access and use of various forms of TRM, particularly, the herbal-based therapies. It was found out that quality control and standards of TRM which rest on the regulatory instruments were not fully prioritized and sometimes neglected. The participants explained that the efficacy and safety of some aspects of TRM cannot be guaranteed because most aspects of TRM lack the necessary clinical tests and checks that are requisite to confirm the effectiveness as well as the safety as observed by Gyasi, Mensah, Yeboah, et al. (2015). Other bottlenecks of greater concern regarding TRM practices include poor financing of traditional health care, indecorous packaging and labelling, lack of expiration and dosage indications, indecent environmental and hygienic conditions of their preparation, and the application of “communal healing” or “cure all concepts”—where a single medicine is purported to cure every kind of ailment. This finding corroborates with other research discoveries (see Addo, 2007; Gyasi, 2014; Gyasi, Mensah, Yeboah, et al., 2015; Sirois, 2008a) which report similar challenges with TRM. These bottlenecks are as a result of poor educational background of and training packages for most of the TMPs. Consequently, the effectiveness, professionalism, and credibility of the TMPs and their practices are compromised. This has implications on the well-being and welfare of the unsuspecting patient. Nevertheless, this finding is inconsistent with Stanifer et al. (2015) who found that lack of monitored dosing, scientific evidence, and diagnostic testing are strong determinants of TRM use (Figure 1).

This is the first pure qualitative study to enrich understanding on factors that stimulate the decision-making regarding TRM use among the adult population in the context of the Ashanti Region of Ghana. This pioneering study has described the impact of potential “pull” and “push” factors in the behaviour of individuals in tandem with TRM utilization. The study has generated discussion towards a better understanding of why people of various socio-economic, cultural, and politico-religious backgrounds consume traditional medical services despite headways of orthodox medical practices. This is nested in the emerged conceptual model which may contribute to further investigations as regards TRM utilization and its associated motivations. The trustworthiness of the study results is preserved by taking cognisance of the confirmability and dependability by recording verbatim and auditing the transcripts and notes during the interview processes. The study notwithstanding has several limitations. These are primarily related to the methodology. The study participants were recruited through purposive and or theoretical sampling which may have the tendency of leaving out some potential respondents with the most accurate and rich experiences regarding TRM use. Ultimately, the study findings may not be generalizable but could only represent the respondents and individuals with similar attributes. A recall bias is inevitable because accounts were given in retrospect over a period of 12 months preceding the interviews.

## Conclusions

This qualitative research provides an insight into the understanding of the mechanisms that motivate individuals to make choices and take actions in the context of TRM consumption. This study has demonstrated that no one single reason is associated with TRM use among the adult population in the Ashanti Region of Ghana. The decision-making towards TRM use is potentially influenced by interwoven and inseparable themes. However, TRM use is more strongly attributed to personal health beliefs which constitute the “pull factors” than to “push factors”—dissatisfaction with orthodox medical practices. TRM use is unlikely to be influenced by aspects of conventional health care; rather, it is a reflective of the personal beliefs of the people and/or groups with ties and relationships. Differences in TRM use between the rural and urban participants are mainly subject to the difficulties inherent in accessing certain forms of health care in rural settings. To maximize the utility of TRM use, efforts are required to deal squarely with the pertinent bottlenecks in the access and use of TRM. This would warrant the patient's safety, welfare, and well-being. Also, the implementation of NHIS ought to be made to cover the TRM practices in Ghana.
